# Co-delivery of metformin and levofloxacin hydrochloride using biodegradable thermosensitive hydrogel for the treatment of corneal neovascularization

**DOI:** 10.1080/10717544.2019.1609623

**Published:** 2019-05-15

**Authors:** Dong Liu, Qianni Wu, Yuqiong Zhu, Yijun Liu, Xiuli Xie, Sihan Li, Haotian Lin, Weirong Chen, Fangming Zhu

**Affiliations:** a GDHPPCLab, School of Chemistry, Sun Yat–Sen University, Guangzhou, China;; b State Key Laboratory of Ophthalmology, Zhongshan Ophthalmic Center, Sun Yat-Sen University, Guangzhou, China;; c Key Lab for Polymer Composite and Functional Materials of Ministry of Education, School of Chemistry, Sun Yat–Sen University, Guangzhou, China

**Keywords:** Thermosensitive hydrogel, corneal neovascularization, metformin, subconjunctival injection, synergistic effect

## Abstract

Corneal neovascularization (CNV) is one of the major causes of severe disorders in ocular surface. Subconjunctival administration provides a localized and effective delivery of anti-angiogenic agents to inhibit neovascularization. In the present study, the ABA triblock copolymer of poly(*D,L*-lactic-*co*-glycolic acid)-block-poly(ethylene glycol)-block-poly(*D,L*-lactic-*co*-glycolic acid) (PLGA-PEG-PLGA) was used as a sustained drug delivery carrier for metformin (MET) and levofloxacin hydrochloride (LFH). Both drugs and PLGA-PEG-PLGA copolymers could be easily dissolved in water at low or room temperature and the mixed solution could form a drug-loaded thermosensitive hydrogel in terms of body temperature response. The *in vitro* release investigation displayed a sustained release of MET and LFH from the formulation for one month. The *in vivo* efficacy of subconjunctival injection of the MET + LFH loaded thermosensitive hydrogel in inhibiting CNV was evaluated on a mouse model of corneal alkali burn. Compared with the single administration of MET or LFH loaded thermosensitive hydrogel, the MET + LFH loaded thermosensitive hydrogel remarkably inhibited the formation of CNV. The sustained release of MET and an antibiotic (LFH) provides synergistic therapeutic outcome. As a result, the co-delivery of MET and LFH using PLGA-PEG-PLGA thermosensitive hydrogel by subconjunctival injection has great potential for ocular anti-angiogenic therapy.

## 1. Introduction

Generally, the normal cornea is an avascular and transparent connective tissue (Chang et al., 2012). However, corneal neovascularization (CNV) is a severe complication induced by various inflammatory, infectious, and degenerative ocular disorders (Roshandel et al., 2018). Besides, CNV represents an important cause of corneal graft rejection and it is also a major reason for corneal opacity and subsequent vision loss or even blindness. Thus, neovascularization of the cornea and other parts of the eye represents an important and worldwide public health problem (Chang et al., 2001; Papathanassiou et al., 2008). Angiogenesis is a complex multistep process induced by multiple factors, such as vascular endothelial growth factor (VEGF), angiopoietin (Ang), basic fibroblast growth factor (bFGF), and other cytokines (Li et al., 2013). Among them, VEGF has been proven to be a major regulator of CNV (Papathanassiou et al., 2008). Anti-VEGF agents including Bevacizumab, Ranibizumab, and Pegaptanib, are effective in inhibiting the growth of new blood vessels and approved by Food and Drug Administration (FDA) for the clinical treatment of neovascularization (Oh et al., 2009). Commercial eye drops or ointments, suffering from extremely low bioavailability (<5% can be absorbed by eye) and rapid clearance mechanisms, are the most common ocular drug delivery systems (Liu et al., 2012). Therefore, repetitive topical drug administration with high dosage is often required to achieve a therapeutic effect, which may lead to inconvenience and severe adverse effects. To overcome the physiological barriers in the eye, intraocular injection is also applied to topical drug delivery. However, frequent intraocular injection also has poor patient compliance, discomfort, and risk of infection. Unfortunately, previous studies demonstrated the short half-lives of anti-VEGF proteins in ocular tissues after intraocular injection, and clinical trials have validated that frequent injections could effectively inhibit neovascularization (Bakri et al., 2007; Agrahari et al., 2016). What’s worse, it will greatly increase the cost of therapy for patients.

Metformin (MET), a typical drug used for the treatment of type 2 diabetes, works by stimulating the adenosine 5’-monophosphate (AMP)-activated protein kinase and influences the glucose uptake from the blood (Evans et al., 2005). A latest breakthrough has revealed that the MET could prevent various chronic diseases including cancers (Stynen et al., 2018). Besides, MET is also a potential agent for inhibiting neovascularization. Tan et al. (2009) indicated that MET increases the antiangiogenic thrombospondin-1 level to inhibit angiogenesis *via* nuclear factor kappa-B (NF*κ*B) pathway. Joe et al. (2015) also found that MET can inhibit VEGF-induced cell proliferation and exhibit anti-angiogenesis effects *via* suppressing the Flk-1 level. In the past decades, a variety of polymeric carriers have been extensively investigated for sustained and controlled drug delivery. Contact lens, implants, polymeric nanoparticles, liposomes, dendrimers, and hydrogel have attracted great attention in the field of ocular drug delivery (Gaucher et al., 2010; Ashaben, 2013). Biodegradable polymers, which can be eventually absorbed by the tissues and eliminate the drawback of subsequent removal, have been widely used as drug carriers, especially for ocular drug delivery (Kimura & Ogura, 2001). Poly(lactic acid; PLA), poly(ε-caprolactone; PCL), poly(trimethylene carbonate; PTMC), and poly(lactide-co-glycolide; PLGA) are the most commonly used biodegradable materials for drug delivery. Among them, PLGA with excellent tissue compatibility, safety profile, and controllable degradation rate, is one of the most potential candidates for ocular drug delivery. Thermosensitive hydrogel or thermogel, which undergoes a reversible sol-gel transition as temperature changing has arisen more and more attention in medicinal application (Zhang et al., 2015). At room temperature (25 °C), the thermosensitive hydrogel is in a sol state and transforms into a gel state with the temperature increased to body temperature (37 °C; Loh et al., 2018). Drugs and therapeutic molecules can be mixed with the polymer/water system at low or room temperature. Then the corresponding formulations are administered into target tissue *via* injection. After injection, the formulations transform into drug delivery gel depots because of the *in-situ* sol-gel transition. (Liow et al., 2016). The drugs incorporated into the network structure of the gel can be released slowly, thus prolonging the biological half-life and reducing side effects and toxicity. Additionally, there are none of organic solvents used throughout the preparation process, leading to a good biocompatibility. Biodegradable thermosensitive hydrogels, especially the poly(lacticacid-co-glycolic acid)-poly(ethylene glycol)-poly(lactic acid-co-glycolic acid) (PLGA-PEG-PLGA) triblock copolymers hydrogel, possessing the advantages of controllable biodegradation rate, adjustable sol-gel transition, good biocompatibility, and effective drug delivery, is one of the most potential biomaterials (Yu & Ding, 2008; Lei et al., 2017; Shang et al., 2017). It has been reported that the PLGA-PEG-PLGA thermosenstive hydrogel is an effective drug carrier for the treatment of various diseases, such as antiosteopenia therapy (Liu et al., 2017), anti-cancer therapy (Shen et al., 2017), anti-capsular formation (Luan et al., 2018), and antidiabetic therapy (Chen et al., 2016). Previous studies also reported the bevacizumab loaded PLGA-PEG-PLGA thermosensitive hydrogel with sustained release properties has been applied to treat posterior segment disorders and has achieved great efficacy (Xie et al., 2015).

In the present study, a thermogelling polymer PLGA-PEG-PLGA was synthesized and used as a drug carrier. On the other hand, considering that the initial inflammatory response is a key factor to induce CNV. Inflammatory cells can produce a great number of pro-inflammatory cytokines and angiogenic growth factors (especially VEGF) under inflammatory condition (Than et al., 2018). Therefore, it is also necessary to effectively inhibit infections and inflammations. We used levofloxacin hydrochloride (LFH), a hydrophilic antibiotic commonly used to treat ocular infections and inflammatory responses, combined with MET to inhibit CNV (Holland et al., 2007; Islan et al., 2016). Combination therapy of multiple drugs at different stages of the ocular diseases can offer a more effective treatment because of their synergistic effects (Jain, 2001; Than et al., 2018). On the basis of the above-mentioned facts, we prepared a localized and long-term co-delivery system of MET and LFH loaded thermosensitive PLGA-PEG-PLGA hydrogel (MET + LFH@Thermogel) to inhibit CNV by subconjunctival administration. CNV was induced by the alkali-burn injury in mice model. Moreover, the prepared MET + LFH@Thermogel was singly subconjunctival injected to evaluate the anti-neovascular effect *in vivo*. Meanwhile, the *in vitro* properties of MET + LFH@Thermogel was also characterized.

## 2. Materials and methods

### 2.1. Materials and animals

Poly(ethylene glycol; PEG, *M*_n_ = 1500), stannous octoate (Sn(Oct)_2_) of purity 95% and LFH were purchased from Sigma-Aldrich (St. Louis, MO). *D,L*-lactide (LA) and glycolide (GA) were provided by Jinan Daigang Biomaterial Co., Ltd. , China) and recrystallized three times with reflux in toluene and dried under a vacuum for 24 h before use. MET was obtained from Selleck Chemicals (Shanghai, China). Proparacaine eye drops (0.5%) and 3% sodium pentobarbital were purchased from Santen Pharmaceutical Co., Ltd., (Osaka, Japan). Human corneal epithelial cells (HCECs) lines were provided by the laboratory of Professor W. Chen (Zhongshan Opthalmic Center, Guangzhou, China). Cell Counting Kit was purchased from Peptide Institute Inc., (Osaka, Japan). Chemicals and reagents for immunofluorescence and hematoxylin-eosin (H&E) staining were all provided by Service Biotechnology Co., Ltd. (Wuhan, China). Distilled water was prepared by the Milli-Q Plus system (Millipore Co., MA). All the chemicals used in this research were of analytical grade and without further purification.

C57BL/6J mice (male and female, 7–8 weeks) were obtained from Guangdong Medical Experimental Animal Center (Guangdong, China). The mice were housed under specific pathogen free (SPF) standard condition (25–28 °C, relative humidity of 40–60%, and light/dark cycle of 12/12 h) in the Animal Experimental Center of Zhongshan Ophthalmic Center, Sun Yat-Sen University, China. The animals were fed with a standard animal food and free access to water. The procedures for animal care and experiments complied with the Association for Research in Vision and Ophthalmology (ARVO) Statement Regarding the Use of Animals in Ophthalmic and Vision Research, and the guidelines of the Animal Experimental Ethics Committee of Zhongshan Ophthalmic Center, Sun Yat-Sen University, China.

### 2.2. Synthesis and characterization of the triblock copolymer

The PLGA-PEG-PLGA triblock copolymers were synthesized through ring-opening polymerization of *D,L*-LA and GA initiated by PEG-OH as macroinitiator under dry nitrogen, with Sn(Oct)_2_ used as catalyst. Briefly, PEG was added into a branched-neck round bottom flask and dried under vacuum with stirring at 130 °C for 4 h. Subsequently, a certain amount of LA, GA, and Sn(Oct)_2_ were added under dry nitrogen. The reaction system was kept at 140 °C under stirring for 12 h. Then, the reaction system was cooled to room temperature under nitrogen atmosphere. The products were washed by 80 °C water to remove soluble low molecular weight polymers. The final product was dried by lyophilization and stored at −20 °C until use.

The chemical structure of the copolymers was determined by ^1 ^H NMR, using a 400 MHz nuclear magnetic resonance (NMR) spectrometer (Bruker Advance III, Varian, Chicago, IL). Deuterated chloroform (CDCl_3_) was used as the solvent and tetramethylsilane (TMS) was used as the internal reference to determine chemical shifts (*δ*). Molecular weight (*M*_W_) and molecular weight distribution (*M*_W_/*M*_n_) of the copolymers were determined using a gel permeation chromatography (GPC) system (Waters 2414, Milford, MA) with a differential refractometer. Tetrahydrofuran was used as a mobile phase at a flow rate of 1 ml min^−1^. Narrow molecular weight distribution polystyrene standards were used for MW calculation.

### 2.3. Preparation of the MET + LFH@thermogel and rheological analysis

A certain amount of PLGA-PEG-PLGA copolymers (0.25 g) were added into stroke-physiological saline solution (SPSS, 1 ml) to obtain 20 wt% polymer/water system at low temperature. Then, a given amount of MET (1, 3, and 5 mg) and 3 mg LFH were completely dissolved in the polymer/water system under ultrasound to form a homogeneous mixed system. The resultant solution was sterilized through a 0.22 *μ*m filter at 4 °C.

The sol-gel transition of the MET + LFH@Thermogel was detected using a HAAKE Rheometric (HMARS III, Thermo, MA) equipped with a 60 mm parallel plate. About 1 ml of polymer/water systems (20 wt % in SPSS) with/without drugs were injected in the parallel plate. The measurements were carried out at a fixed oscillatory frequency of 10 rad/s with a heating rate of 0.5 °C/min from 10 to 40 °C. Storage-(*G*’) and viscous modulus (*G*’’) were recorded as a function of temperature.

### 2.4. *In vitro* drug release and cytotoxicity study

The drug loaded polymer/water system (1 g) was injected into a dialysis bag (MWCO 14000, Spectrapor, CA) and a centrifuge tube containing the dialysis bag was incubated in an air bath at 37 °C. After the formation of *in situ* hydrogels, 10 ml of phosphate buffered saline (PBS, pH 7.4) was added into the centrifuge tube as the release medium. The shaking rate was 80 rpm. At predetermined time intervals, the release medium was withdrawn and replaced by the same amount of fresh PBS. The amount of released MET and LFH was determined by high performance liquid chromatography (HPLC) system (Waters1525) with a C18 column (4.6 mm × 250 mm-5 *μ*m, Agilent ZORBAX Eclipse XDB, CA). For MET, the mobile phase was composed of methanol and 0.02 M phosphoric acid (50: 50, v/v) at a flow rate of 1 ml/min and the temperature of 30 °C. And for LFH, the mobile phase was composed of acetonitrile and 0.02 M phosphoric acid (20: 80, v/v) at a flow rate of 1 ml/min and the temperature of 40 °C. The accumulated release amount was calculated by the calibration curve.

The *in vitro* cytotoxicity of the PLGA-PEG-PLGA thermosensitive hydrogel and MET + LFH@Thermogel with different concentrations of MET (1, 3, and 5 mg/ml) and the same concentration of LFH (3 mg/ml) were evaluated *via* Cell Counting Kit-8 (CCK-8) assay. HCECs(2.5 × 10^4^) were cultured on 24-well plates at 37 °C with 5% CO_2._ The polymer/water systems loaded with/without drugs (0.1 ml) were added into transwell chambers and transformed into *in situ* gels at 37 °C. After 24 h incubation, the cells were washed with PBS, then the transwell chambers containing different concentrations of MET + LFH@Thermogel and PLGA-PEG-PLGA thermosensitive hydrogel were added to the 12-well plates. After another 24 h incubation, the medium was removed and the cells were washed with PBS and 10 *μ*l of CCK-8 solution was added to the 24-well plates and reincubated at 37 °C for 1 h. The assay was performed by a microplate reader (Synergy H1, BioTek Instruments, Winooski, VT ) at 450 nm to measure cell viability, and the cells without any treatment were served as the blank control.

### 2.5. Alkali-burn injury induced CNV in mice

**Figure 1.**
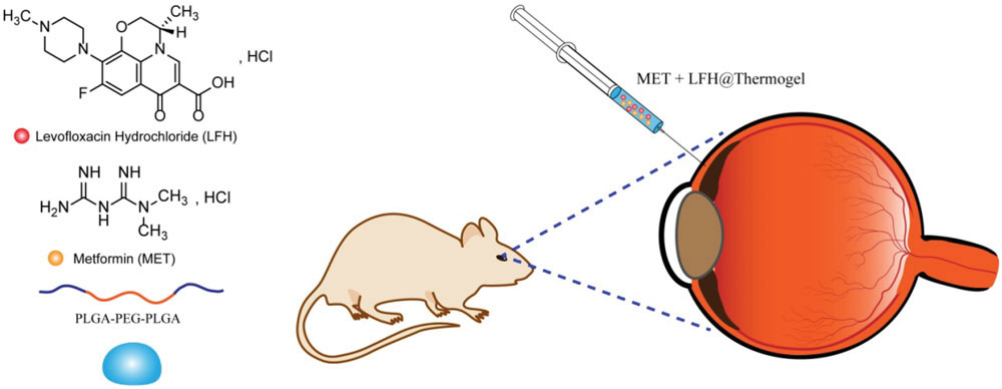
The molecular structures of MET and LFH and schematic diagram of subconjunctival injection of the MET + LFH@Thermogel.

All procedures and tests were performed under general anesthesia by intraperitoneal injections of 3% pentobarbital (1 ml/kg) and topical administrations of 0.5% proparacaine. Alkali-burn injury to the cornea was induced by placing a sterilized filter paper (2 mm in diameter) soaked with 1 N sodium hydroxide solution for 30 s. Then, the ocular surface and conjunctival fornixes were extensively flushed with 10 ml of sterilized PBS solution. One day after alkali-burn injury, CNV were imaged under a slit lamp microscope (SL-D7/DC-3/IMAGEnet, Topcon, Japan). Subsequently, the CNV mice were randomly divided into 4 groups, the first group received single subconjunctival injections of PLGA-PEG-PLGA thermosensitive hydrogel without drugs, the second group received single subconjunctival injections of LFH loaded thermosensitive hydrogel (LFH@Thermogel); the third group received single subconjunctival injections of MET loaded thermosensitive hydrogel (MET@Thermogel); and the fourth group received single subconjunctival injections of MET + LFH@Thermogel (Figure 1).

### 2.6. Visualization and quantification of CNV

Seven days after alkali-burn injury, corneas were imaged by the slit lamp microscope (SL-D7/DC-3/IMAGEnet, Topcon, Japan) and the CNV areas were analyzed using ImageJ 1.40 image analysis software (NIH, Bethesda, MD). According to the previous study, the vessel area (VA) was calculated by the using Equation (1) (Rogers et al., 2007):

(1) VA=0.02π×VL×CH

where the VL is vessel lengths measured from the limbus to the leading edges and the CH is the sectoral circumference, which was expressed as clock hours (1 being 30 degrees of arc).

### 2.7. H&E staining and immunofluorescence evaluation

Mice were euthanized after seven days and then the excised corneas were imbedded in paraffin and sectioned to 4 *μ*m-thickness. The sections were stained by H*&*E and examined under a light microscope (Axiophot, Zeiss, Germany). To analyze the expression of F4/80 and VEGF in corneas, the sections from paraffin-embedded corneas were deparaffinized, then they were exposed to ethylenediaminetetraacetic acid (EDTA) antigen repair buffer (pH 8) for antigen retrieval and blocked in albumin from bovine serum (BSA). The tissues were incubated with a rabbit polyclonal anti-F4/80 antibody and a goat polyclonal VEGF antibody separately overnight at 4 °C, then the sections were incubated with fluorescently labeled secondary antibody (Goat Anti-Rabbit IgG H&L (Alexa Fluor^®^ 647) or Donkey Anti-Goat IgG H&L (Alexa Fluor^®^ 647)). Subsequently, the tissues were incubated with a 4’,6-diamidino-2-phenylindole (DAPI) solution and analyzed under fluorescence microscope (Axioplan2 Imaging, Zeiss, Germany).

### 2.8. Quantitatively assay of cytokines by luminex^®^ xMAP^®^ technology

On the seventh day after alkali-burn injury, mice were euthanized and the corneas were carefully enucleated, then the proteins were extracted using cell lysis buffer with the addition of protease inhibitors. The expression levels of mice interleukin-1*β* (IL-1*β*), IL-6, IL-17, Ang-2, VEGF-A, VEGF-C, and tumor necrosis factor (TNF-*α*) were quantified by Milliplex MAP Rat Cytokine/Chemokine Magnetic Bead Panel (Meck Millipore, Billerica, MA) and Luminex^®^ xMAP^TM^ technology (Luminex Corporation, Austin, TX), the data were analyzed by MILLIPLEX^®^ Analyst 5.1 software (Meck & Co Inc., Kenilworth, NJ) .

### 2.9. Statistical analysis

All experiments were performed in triplicate. The data were statistically analyzed using SPSS Statistic 17.0 software (SPSS Inc, Chicago, IL). The data were expressed as the mean ± standard error. A statistically significant difference was shown when *p* < .05 (*), while *p* < .01 (**) and *p* < .001 (***) were considered as highly significant.

## 3. Results and discussion

### 3.1. Synthesis and characterization of the PLGA-PEG-PLGA triblock copolymers

**Figure 2.**
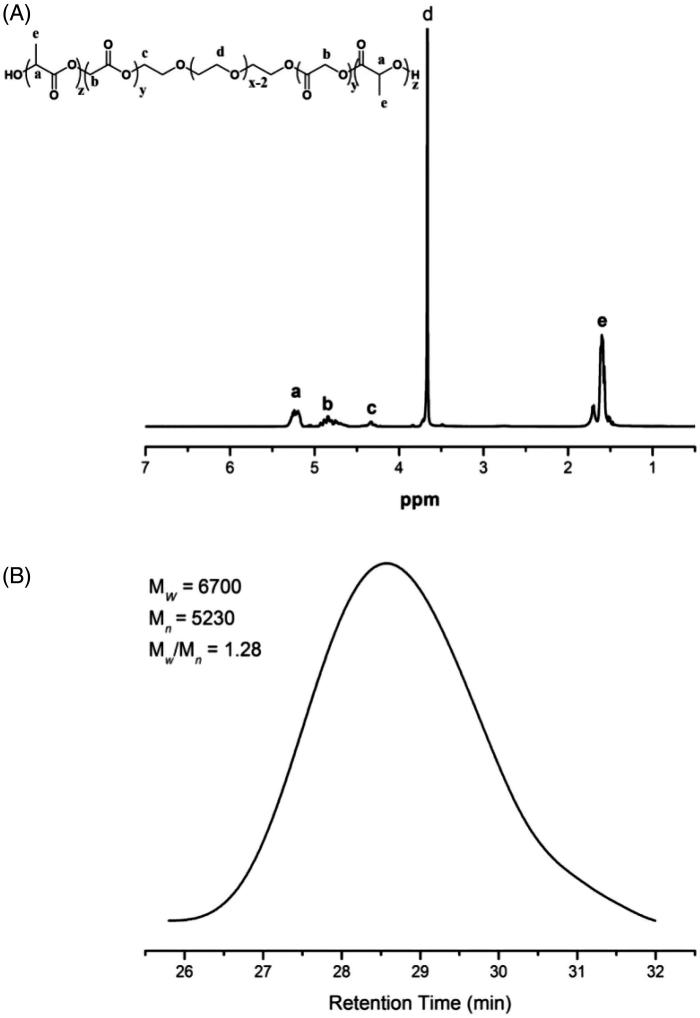
H NMR spectrum (A) and GPC curve (B) of PLGA-PEG-PLGA triblock copolymer.

The ^1 ^H NMR spectrum and GPC curve of PLGA-PEG-PLGA copolymer are shown in Figure 2. The signals appeared at 3.6 ppm were attributed to –CH_2_–in PEG block. The chemical shifts at 5.2 and 1.6 ppm were assigned to –CH– and –CH_3_ in the LA unit, the 4.8 ppm was –CH_2_– in the GA unit, and the 4.2 ppm was –CH_2_– in ethylene glycol neighboring to the GA unit. Based on the calculation results of ^1 ^H NMR spectrum, the block weight ratio of PLGA:PEG:PLGA was about 6:5:6. The GPC results showed the weight-average molecular weight (*M*_w_) and *M*_w_/*M*_n_ of the triblock copolymer were 6700 and 1.28, respectively. 

### 3.2. Sol-gel transition of the MET + LFH@thermogel

**Figure 3.**
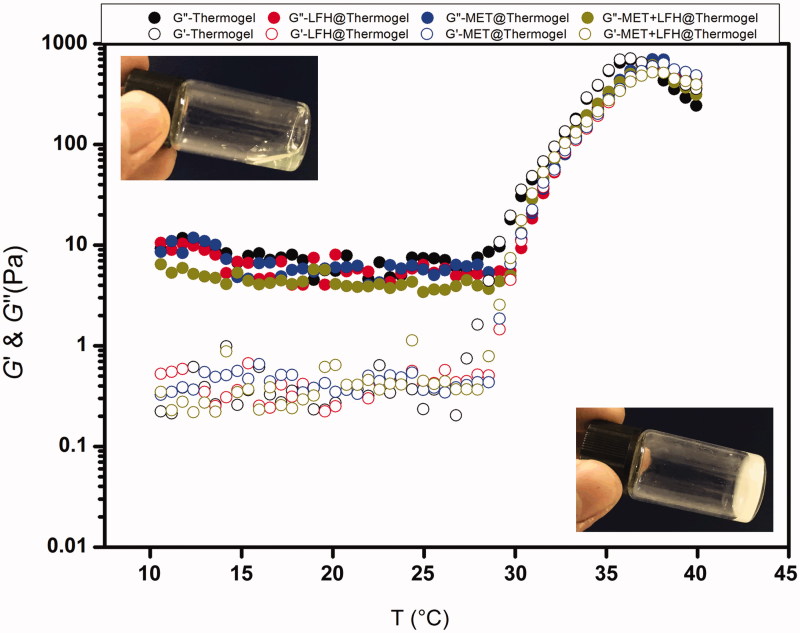
Storage modulus (*G*’) and loss modulus (*G*”) of the drug loaded polymer/water system as a function of temperature. The concentrations of MET and LFH were both 3 mg/ml. The inserted pictures show the sol state of the MET + LFH@Thermogel at room temperature (25 °C) and gel state of the MET + LFH@Thermogel at body temperature (37 °C).

Dynamic rheological measurements were performed to assess the changes in *G*’ and *G*’’ of the thermosensitive hydrogels loaded with/without drugs. As shown in Figure 3, the PLGA-PEG-PLGA thermosensitive hydrogel, LFH@Thermogel, MET@Thermogel, and MET + LFH@Thermogel all exhibited a sol-gel transition with the increase of temperature. *G*’ represents the elastic behavior or the elastic component of the complex shear modulus, whereas *G*’’ represents the viscous behavior. The *G*’ values were lower than *G*’’ at low or room temperature, demonstrating the mixed solution was free-flowing and suitable for injection. With the increase of temperature, both *G*’ and *G*’’ increased, and *G*’ was equal to *G*’’ at approximately 31 °C. The sol-gel transition was observed by naked-eye as well. After the formation of *in situ* thermosensitive hydrogel, conversely, the *G*’’ was lower than *G*’, and both *G*’ and *G*’’ reached maximum near physiological temperature of 37 °C. Moreover, there was no obvious difference in rheological behavior between the four groups, indicating that the addition of MET and LFH did not affect the sol-gel transition of the thermosensitive hydrogel. It is beneficial for ocular drug delivery of such formulations.

### *3.3.* In vitro *drug release and cytotoxicity*

**Figure 4.**
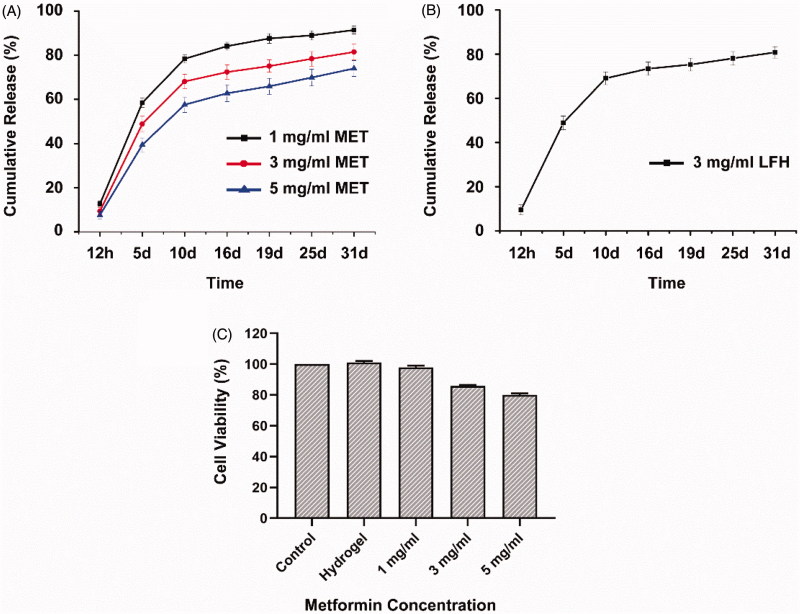
(A) *In vitro* release profiles of MET from MET + LFH@Thermogel with various drug loading amounts in PBS. The results are presented as mean + SD (*n* = 3). (B) *In vitro* release profile of LFH from MET + LFH@Thermogel in PBS and LFH concentration was 3 mg/ml. The results are presented as mean ± SD (*n* = 3). (C) *In vitro* cytotoxicity of the thermosensitive hydrogel and MET + LFH@Thermogel (MET concentrations ranged from 1–5 mg/ml and LFH concentration was 3 mg/ml) against HCECs after 24 h incubation. The results are presented as mean ± SD (*n* = 3).

The *in vitro* drug release profiles of MET + LFH@Thermogel were displayed in Figure 4(A,B) . All the three MET + LFH@Thermogel formulations presented a long-term release pattern for one month and Figure 3(B) showed more than 20% of LFH was released from the hydrogel matrix in the initial 48 h, which provided a sufficient drug dosage for suppressing infection and inflammation at the initial stage of CNV. At the same time, the release of MET maintained a sustained manner to inhibit the growth of new blood vessels. Since the manufacturing process ensured almost no loss of drugs, the encapsulation efficiency was approximately 100%. The release curves showed the higher the drug loading amount, the lower the cumulative release percent. This is because that the cumulative release percent is the ratio of the drug release amount to loading amount. Although the release amount increases, the drug loading amount becomes larger and the ratio of them decreases (Gong et al., 2009; Luo et al., 2016; Sun et al., 2017). Previous studies also indicated that the drug release from the biodegradable hydrogel due to both the drug diffused from the porous structure of hydrogel and the polymer degradation (Tang et al., 2008; Xie et al., 2015). The *in vitro* release profile demonstrated the MET + LFH@Thermogel can provide an extended and sustained drug release for the treatment of ocular disorders.

The *in vitro* cytotoxicity of PEG-PLGA-PEG thermosensitive hydrogel and MET + LFH@Thermogel with different drug concentrations was evaluated *via* CCK-8 assay. After incubation of the PEG-PLGA-PEG hydrogel for 24 h, the cell viability was approximately 100%, indicating that the thermosensitive hydrogel exhibits very low cytotoxicity and good biocompatibility. With the MET concentration increasing from 1–5 mg/ml, the cell viability of MET + LFH@Thermogel decreased from 96 to 80%. This decreased trend was attributed to the high concentration of MET and the cell viability was dependent on drug concentration. These results implied that the MET + LFH@Thermogel with long-term release and good biocompatibility is appropriate for ocular drug delivery.

### 3.4. Anti-angiogenic effect of MET + LFH@thermogel in alkali-burn injury induced mice CNV model

**Figure 5.**
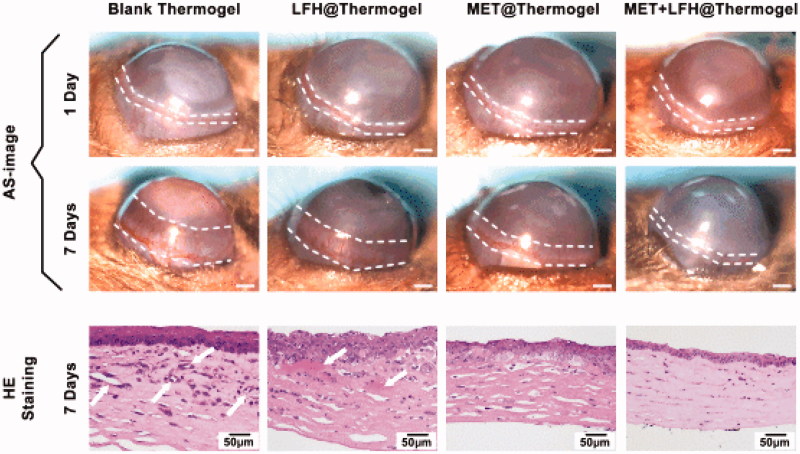
Inhibition of CNV by different treatments in alkali burn induced corneal injury model. (A) Representative cornea images of one day after alkali burn and seven days after alkali burn with different treatment. (B) Representative H&E staining at seven days after alkali burn of different treatment (scale bar = 50 *μ*m). White arrows point out the neovessels.

The thermosensitive hydrogel containing 3 mg/ml of MET and 3 mg/ml of LFH was selected as appropriate concentration for *in vivo* evaluation, which was based on the considerations of drug dosage required to inhibit CNV and anti-infection, the *in vitro* drug release profile and the *in vitro* cytotoxicity of MET + LFH@Thermogel. The drug concentrations of LFH@Thermogel and MET@Thermogel were 3 mg/ml as well. The anti-angiogenic effect of conjunctival injection of LFH@Thermogel, MET@Thermogel, and MET + LFH@Thermogel were first evaluated on mice with CNV. As shown in Figure 5(A), we could observe the representative cornea images from each treated group at seven days after alkali-burn. Since alkali burn causes the damage of corneal epithelial and induction of CNV, quantitative analysis of the CNV areas in the blank control group, LFH@Thermogel, MET@Thermogel, and MET + LFH@Thermogel injected group were performed, indicating CNV areas to be 1.13 ± 0.29, 0.66 ± 0.25, 0.39 ± 0.22, and 0.13 ± 0.11 mm^2^, respectively. The MET + LFH@Thermogel group markedly reduced the area of CNV more than the other groups. Mice treated with MET + LFH@Thermogel led to ∼88% reduction in CNV area compared with blank control group. The efficacy of local ocular drug delivery using thermosensitive hydrogel is much better than topical administration and conventional intraocular injection (Urtti, 2006; Gaudana et al., 2010), since the long-term drug release could efficiently inhibit the outgrowth of immature blood vessels. In comparison with the CNV areas of blank control group, MET@Thermogel treated group, and LFH@Thermogel treated group, the co-delivery of MET and LFH could offer an obvious inhibitory effect on the development of CNV. Figure 5(B) showed the histological changes by H&E staining at seven days after injury, the neovessels could be obviously observed in the blank control group and LFH@Thermogel treated group, and the corneas appeared edema and inflammatory response as well. In contrast, there were no obvious neovessels and a reduction of edema and inflammatory response in the MET + LFH@Thermogel treated group, indicating that the combination therapy of MET and LFH has a much stronger inhibitory effect on CNV and inflammatory response.

**Figure 6.**
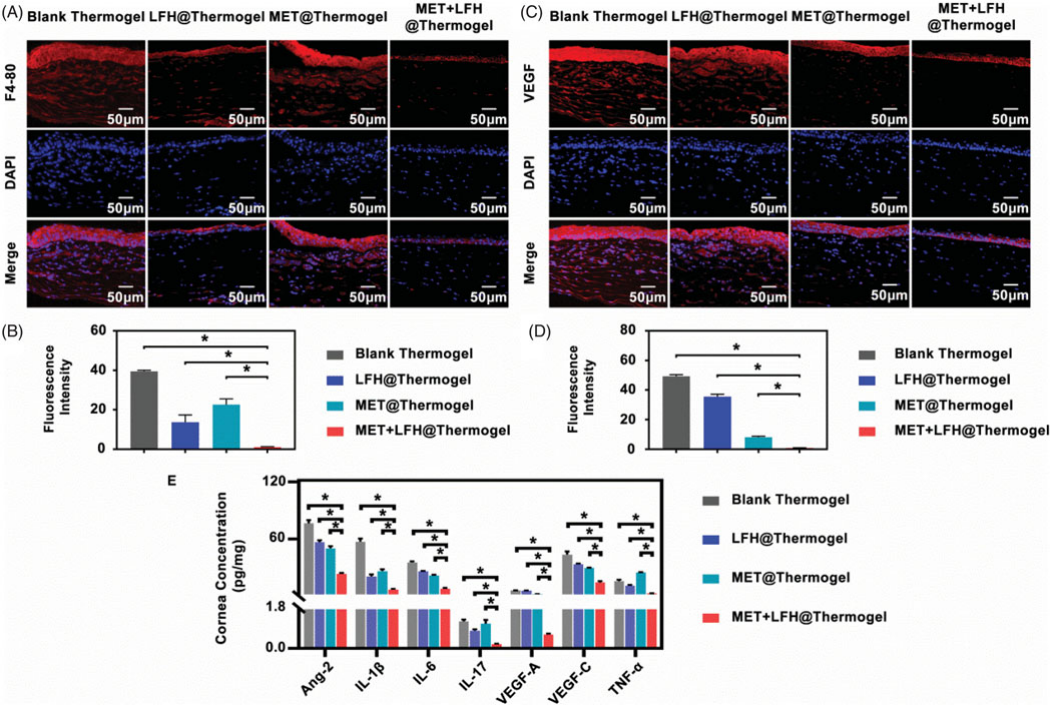
Anti-angiogenic assessment of different treatments on seventh day after alkali burn. (A) Immunofluorescence staining of the cornea with a specific macrophage maker-F4/80 antibody (red color) and (B) the quantitative analysis (scale bar = 50 *μ*m). The results are presented as mean ± SD (*n* = 6). *p < .05. (C) Immunofluorescence staining of VEGF (red color) in the cornea and (D) the quantitative analysis (scale bar = 50 *μ*m). The results are presented as mean ± SD (*n* = 6). *p < .05. (E) Concentrations of Ang-2, IL-1*β*, IL-6, IL-17, VEGF-A, VEGF-C, and TNF-α in the corneas. The results are presented as mean ± SD (*n* = 6). *p < .05.

To further characterize the effect of MET + LFH@Thermogel in corneal neovascularization, immunofluorescence staining for cornea tissues was analyzed. As previously mentioned, the initial inflammatory is a key factor to trigger CNV. Thus, corneal sections were stained with F4/80 because the macrophage marker F4/80 has been extensively used to characterize tissue macrophages (Gordon et al., 2011). As shown in Figure 6(A), the corneas treated with MET + LFH showed significantly fewer infiltrating F4/80-positive macrophages than the other three treated groups (*p* < .05). Besides, VEGF staining was also analyzed to demonstrate the anti-VEGF effect of MET + LFH@Thermogel. Although MET alone suppressed VEGF to a lesser extent (Figure 6(C)), MET + LFH@Thermogel showed most significant suppression on the expression of VEGF than the other three groups (*p* < .05). Previous studies revealed that macrophages play an important role in neovascularization by secreting matrix metallopreoteinase-2 (MMP-2), MMP-7, MMP-9, MMP-12, and cycloocygenase-2 (COX-2; Kale et al., 2014; Liu et al., 2015). Lu et al. (2009) also found that the interleukin-1 receptor antagonist (IL-1R*α*), expressed by macrophage, can regulate the expression of VEGF by binding IL-1R. Factors related to the biological behavior of macrophages during inflammation may affect the extent of CNV (Bainbridge 2007). Therefore, the effective inhibition of the initial inflammatory response is helpful to anti-neovascularization. The immunofluorescence analysis indicated the treatment of MET + LFH@Thermogel showed significant suppression on the number of infiltrating F4/80 macrophage and the expression of VEGF in corneas. This is due to the synergistic effect of antibiotic and anti-neovascularization agent. The release of LFH mainly suppresses infection and inflammation with limited anti-angiogenic effect, while the release of MET effectively inhibits neovascularization with limited anti-inflammatory effect. The co-delivery of MET + LFH leads to a much better therapeutic effect on CNV.

We further detected the concentrations of inflammatory and angiogenic cytokines in corneas after treatment, including Ang-2, IL-1*β*, IL-6, IL-17, VEGF-A, VEGF-C, and TNF-α. As shown in Figure 6(E), the co-delivery of MET and LFH produced the most significant effect on reducing all the cytokine levels in corneas compared with the other two groups (*p* < .05). LFH expressed strong inhibitory effect on inflammatory cytokines levels (IL-1*β*, IL-17, and TNF-α), although MET has a certain inhibitory effect on the expression of IL-6. The inflammatory cytokines levels in MET + LFH@Thermogel treated group were significantly lower than those in LFH@Thermogel treated group (*p* < .05). This result indicated that the treatment of MET + LFH@Thermogel has the most significant effect on reducing the inflammatory cytokines, which is consistent with our results of macrophage infiltration shown in Figure 6(A). Low expression levels of inflammatory cytokines help to inhibit the development of CNV. On the other hand, for angiogenic cytokines, MET inhibited the expression levels of Ang-2, VEGF-A, and VEGF-C. It might be attributed to the MET, which increases the antiangiogenic thrombospondin-1 (TSP-1) and decreases the level of VEGF receptor Flk-1 as well (Tan et al., 2009; Joe et al., 2015). Previous studies indicated that the TSP-1 is a potent endogenous inhibitor. It participates in maintaining corneal avascularity and binds to antiangiogenic receptor CD36 to inhibit CNV (Akhurst 2004; Cursiefen et al., 2004; Mwaikambo 2006). The increase of Flk-1 expression is an important inducement for the formation of CNV after alkali burn (Qazi et al., 2009). MET can block the signal transduction pathway of VEGF by inhibiting the expression of Flk-1 (Soraya et al., 2012; Joe et al., 2015). Besides, MET has been proven that it can protect the integrity of epithelial cells under stress conditions, such as inflammation, infection, and hypoxia (Aznar et al., 2016). Taken together, combinational therapy of MET and LFH using biodegradable thermosensitive hydrogel can offer an enhanced inhibitory effect on CNV. With the ability to overcome the ocular surface barriers for localized delivery, MET + LFH@Thermogel produces high bioavailability and effective ocular drug delivery. In addition, the good therapeutic effect is benefited by the synergistic effect of multiple drugs. The demonstrated MET + LFH@Thermogel, which injects biodegradable drug carrier for localized, long-term, and efficient ocular drug delivery, can reduce the injection frequency, thus improving patient compliance and lowering the risk of complications.

## 4. Conclusion

In this study, we developed a sustained co-delivery system of MET and LFH using thermosensitive PLGA-PEG-PLGA hydrogel by subconjunctival injection to inhibit CNV. The free-flowing drug loaded polymer/water system could be prepared by simply mixing at low or room temperature and it exhibited a sol-gel transition to form a drug delivery gel depot at body temperature. The prepared MET + LFH@Thermogel exhibited a sustained drug release *in vitro* for one month and the drug release profile was able to be modulated by the drug loading amount. *In vitro* evaluation of cytotoxicity confirmed the low toxic and good biocompatibility. Moreover, *in vivo* results further demonstrated the sustained co-delivery of MET + LFH reduced the neovascularization area and suppressed the expression levels of inflammatory and angiogenic cytokines, indicating that such a system is more effective in the treatment of neovascularization. In summary, this novel MET + LFH@Thermogel system, which provides a localized, efficient, and cost-effective manner, has great potential for the treatment of ocular neovascularization.
